# Influence of *Eimeria* spp. and *Clostridium perfringens* Infection on Growth Performance and Toltrazuril Residues in Chickens

**DOI:** 10.3390/ani15020216

**Published:** 2025-01-15

**Authors:** Konrad Pietruk, Jacek Karamon, Piotr Jedziniak, Stanisław Tokarzewski, Małgorzata Olejnik

**Affiliations:** 1 Department of Pharmacology and Toxicology, National Veterinary Research Institute, 57 Partyzantów Avenue, 24-100 Puławy, Poland; piotr.jedziniak@piwet.pulawy.pl; 2 Department of Parasitology and Invasive Diseases, National Veterinary Research Institute, 57 Partyzantów Avenue, 24-100 Puławy, Poland; jacek.karamon@piwet.pulawy.pl; 3 Avi Expert—Specialist Veterinary Clinic for Bird Diseases, Gajowa 1, 20-827 Lublin, Poland; aviexpert.lecznica@gmail.com; 4 Department of Basic and Preclinical Sciences, Faculty of Biological and Veterinary Sciences, Nicolaus Copernicus University in Torun, 11 Gagarina Street, 87-100 Toruń, Poland; molejnik@umk.pl

**Keywords:** *Clostridium perfringens*, coccidiosis, *Eimeria*, residue, toltrazuril

## Abstract

Coccidiosis and necrotic enteritis are among the most common diseases affecting poultry, with economic impact due to reduced production and the costs of treatment and prevention. Eimeria invasion contributes to gut damage that promotes the growth of other harmful pathogens, such as *Clostridium perfringens*. Coccidiostats, with toltrazuril as an example, are widely used to control these infections. In this study, we assessed the effects of *Eimeria* spp. infection and coinfection with *Eimeria* spp. and *Clostridium perfringens* on chicken weight and toltrazuril concentrations, along with two metabolites—toltrazuril sulfoxide and toltrazuril sulfone—using liquid chromatography coupled with mass spectrometry. The analysis of liver and muscle samples showed lower levels of residues in groups challenged with *Eimeria* spp. In the case of combined *Eimeria* and *C. perfringens* infection, no significant differences were observed. These findings suggest that the combination of *Eimeria* and *C. perfringens* does not significantly alter the residue levels of toltrazuril and its metabolites, providing valuable insight into the pharmacokinetics of toltrazuril in poultry under infection conditions. This research contributes to optimizing drug use and residue management in poultry production.

## Simple Summary

Coccidiosis is a disease caused by parasites called *Eimeria* that infect chickens. These organisms damage the intestines, making it harder to digest food and absorb nutrients. This can also lead to further problems like infection from harmful bacteria. Coccidiosis is a major issue for the poultry industry which causes costs of millions of dollars annually. To control coccidiosis, farmers use anticoccidial drugs like toltrazuril. This drug works well by killing the parasites. However, after using toltrazuril, there is a period of time that need to be fulfilled to ensure that animal products are safe to eat. When chickens are sick, their bodies may process drugs differently. This can affect how the drug works and how long it stays in their system. The results of this study confirm the impact of parasites on growth performance, evidenced by a 30% reduction in body weight, as well as a lower concentration of toltrazuril sulphone residues in liver and muscle samples.

## 1. Introduction

Coccidiosis is a disease caused by protozoal parasites belonging to the Eimeria genus. Seven species specifically infect domestic chicken (*Gallus gallus domesticus*): *E. acervulina*, *E. maxima*, *E. mitis*, *E. preacox*, *E. brunetti*, *E. necatrix*, *E. tenella*, *E. havani*, and *E. mivati* [1,2]⁠. Eimeria infection can cause severe damage to mucosal cells, increasing cell permeability, plasma protein leakage, and impaired digestion [3]. The negative influence of coccidian infection interferes with gut microbial communities in the gastrointestinal tract (GIT), which can lead to dysbiosis [4]. These changes can promote the colonization and proliferation of other pathogens, such as *Clostridium perfringens* or *Salmonella enteritidis* [5]. Coccidiosis is considered one of the most important factors predisposing to necrotic enteritis due to lesions caused by parasite activity [6,7]. The cost of control, treatment, and lost production caused by necrotic enteritis is estimated to be over USD 2 billion annually [8]. Eimeria infection affects the ability of chickens to assimilate nutrients from digested food by reducing bacterial species such as *Ruminococcaceae* [9]. Structural damage to the intestines by pathogens and dysbiosis can interfere with drug absorption and metabolism. Research has proven that the microbiome in the gastrointestinal tract can affect drug metabolism [10,11].

Unfortunately, prophylactic measures can be inefficient, which could lead to a clinical form of coccidiosis [12]. Coccidiostats are the most common and efficient therapeutic agents used to prevent and control coccidiosis. Pharmacologically active substances are authorized to be used as feed additives or classical veterinary drugs. Feeds containing particular coccidiostats can be used as a preventive factor in the early stages of poultry husbandry until the last day of slaughter. When the clinical form of coccidiosis occurs, the most common procedure is to administer anticoccidial veterinary drugs. Toltrazuril is one of the most popular coccidiostats used when dealing with the clinical form of coccidiosis. Applying a dosage of 7 mg/kg per body weight was revealed to be effective against all species of Eimeria relevant to chickens [13]. The combination of water solubility, activity against all intracellular stages of *Eimeria* spp., and no effect on the development of natural immunity against coccidia make toltrazuril a very effective anticoccidial agent [14]. Studies on the pharmacokinetics of toltrazuril revealed that toltrazuril sulfon is the main metabolite and was set as a marker residue [15]. To ensure the safety of consumers of products of animal origin, a withdrawal time of 16 days for poultry is required with maximum residue levels as follows: muscle—100 µg/kg; skin and fat—200 µg/kg; liver—600 µg/kg; kidney—400 µg/kg; and eggs—140 µg/kg [16,17].

Kinetics and depletion studies are typically performed in healthy animal populations, with the assumption that the pharmacokinetic data obtained will apply to diseased animals. Disease states, particularly those involving the gastrointestinal tract, can profoundly influence the absorption, distribution, metabolism, and elimination (ADME) processes of drugs [18]. Alterations in these pharmacokinetic processes may, in turn, impact critical factors such as the withdrawal period and the effective dosage of a drug. Such interactions between disease and drug kinetics have been described in several studies focusing on antibiotics. For example, Bladek and Gbylik Sikorska [19,20] highlighted that the presence of disease can significantly alter the elimination rates and residual depletion of drugs, leading to discrepancies in expected drug residues and withdrawal times.

Building on this understanding, the objective of the current study was to evaluate the impact of *Eimeria* spp. infection, both as a singular infection and in combination with Clostridium perfringens, on drug kinetics. Specifically, this study aimed to assess how these infections affect the growth performance of animals and the residue levels of toltrazuril and its metabolites in liver and muscle tissues.

## 2. Materials and Methods

### 2.1. Experimental Design

The experiment was performed according to Approval No. 81/2019 of the Local Ethics Committee of Animal Experimentation in Lublin. A total of 130 one-day-old chicks of Rosa 2 were obtained from a local hatchery. The chickens were randomly divided into six groups (Table 1), and a 21-day acclimatization period was conducted. Birds had free access to water and feed. The pre-starter, starter, and grower feeds were composed of a min of 21%, 19.5%, and 18.5% crude protein and a min of 3.0%, 4.5%, and 6.0% crude fat with 2950 kcal/kg, 3020 kcal/kg, and 3125 kcal/kg metabolizable energy, respectively. Chickens were fed with pre-starter feed from 0 to 10 days of life, starter from 10 to 19 days, and grower from 19 days to the end of the experiment. All birds were maintained in a temperature-controlled environment in cages (each group separately) suitable for their requirements. The room was maintained at 20–30 °C and lit for 16 h daily. The temperature was adjusted according to the growth of chickens—with higher temperatures set at the beginning of the experiment and lower with birds getting older.

On the 22nd day (day 1 of the experiment), chickens from groups B, C, E, and F were infected with *Eimeria* spp. by administering 2 mL of a suspension containing 2.5 × 10^4^ oocysts by oral gavage. The coccidia suspension consisted of *E. tenella* 47%, *E. maxima* 30%, *E. acervulina* 17%, and *E. necatrix* 6%. The concentration of the oocyst suspension was determined by microscopic examination using a Fuchs-Rosenthal chamber. Individual Eimeria species were evaluated microscopically based on the morphological features of the oocysts, determining their percentage in the suspension [21]. On day 4, birds from groups C and F were coinfected with *Clostridium perfringens* bacteria by administering 1 mL of a solution containing 1.0 × 10^9^ colony-forming units (CFUs) by oral gavage. The calculation was performed using the ISO 15213-2:2023 norm [22]. On days 5 and 6 (post-infection), animals from groups D, E, and F were treated with toltrazuril. Medication was performed according to the label: 1 mL of Baycox 2.5% (Bayer) was added to 1 mL of drinking water (to achieve a dosage of 7 mg/kg per body weight), which was available for 48 h (after 24 h, fresh medicated water was prepared), and after medication, freshwater was reintroduced.

**Figure 1:**
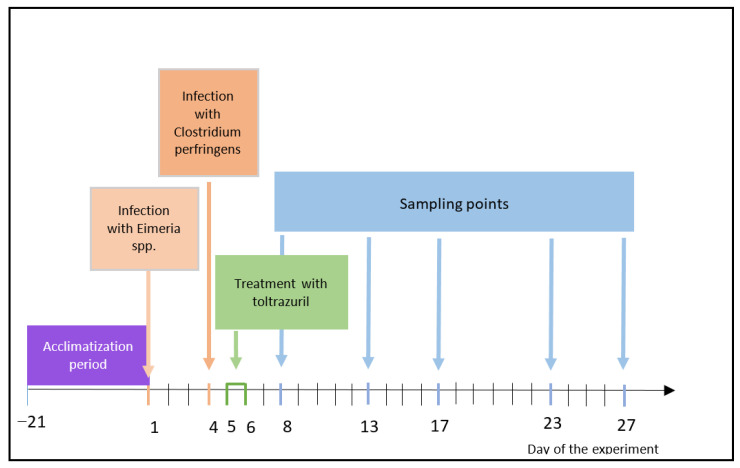
Timeline of the animal experiment.

On days 8, 13, 17, 23, and 27 of the experiment (which corresponds to days 4, 9, 13, 19 and 23 post-infection), five randomly chosen birds from groups D, E, and F were sacrificed by performing a percussive blow to the head, and then liver and muscle samples were collected to analyze residues of toltrazuril and its metabolites. The sampling scheme was set to resemble sampling in the Toltrazuril Summary Report prepared by the European Medicinal Agency [16]. Animal group details and the timeline of the experiment are presented in Table 1 and Figure 1.

### 2.2. Growth Performance

The growth performance was estimated by weighing chickens using a Radwag (Radom, Poland) analytical scale with a precision of 0.1 g. Weighing was performed during sampling points. The mean value of 5 birds from each group on each sample collection day was used as a data point. The data were analyzed using a one-way ANOVA with Tukey’s HSD test to evaluate the differences among the experimental groups.

### 2.3. Determination of Toltrazuril and Metabolites in Liver and Muscle Samples

#### 2.3.1. Sample Preparation

A sample of 2.00 ± 0.01 g of liver or muscle was weighed into a 15 mL polypropylene centrifuge tube and briefly vortex-mixed. Extraction was performed using 5 mL of a 90% aqueous solution of acetonitrile. The sample was vortex-mixed for 1 min and centrifuged at 3500 rpm for 15 min. The supernatant was then defatted using two portions of 3 mL of hexane. The upper layer was transferred into a Falcon tube (Corning, Glendale, AZ, USA) containing 600 mg of C18 sorbent (Agilent, Santa Clara, CA, USA) and 80 mg of PSA sorbent (Merck, Darmstadt, Germany). After vortexing and centrifuging, an additional clean-up step was performed by storing the sample at −20 °C for 1 h. After freezing, the upper layer was collected and evaporated to dryness in a stream of nitrogen at 40 °C. The dry residue was dissolved in 250 µL of 50% aqueous acetonitrile solution, transferred to 1.5 mL Eppendorf tubes (Eppendorf, Hamburg, Germany), and centrifuged at 14,500 rpm for 15 min. Finally, the supernatant was transferred to an HPLC vial with a glass (Agilent, Santa Clara, CA, USA) insert and analyzed using LC-MS/MS (Shimadzu, Kyoto, Japan).

#### 2.3.2. Mass Spectrometry

The analysis was performed on a Shimadzu LCMS 8050 triple quadruple detector using electrospray ionization (ESI) (Shimadzu, Kyoto, Japan) controlled by LabSolution 5.60 SP2 software. The parameters of the mass spectrometer were as follows: nebulizing gas (nitrogen) flow: 2 L min^−1^; heating gas and drying gas (air) flow; 10 L min^−1^ each; interface temperature: 300 °C; temperature of desolvation line: 250 °C; heat block temperature: 400 °C; and capillary voltage: −3 kV for negative ionization. The selected reaction monitoring (SRM) mode was applied with the following *m*/*z* transitions (qualitative and quantitate): toltrazuril: 424.0 to 42.0 and 91.1; toltrazuril sulfone: 455.9 to 42.0 and 339.0; and toltrazuril sulfoxide: 439.8 to 42.0 and 371.0.

#### 2.3.3. Quality Assurance of the Results

To ensure the validity of the results for analyzing residues of toltrazuril and its metabolites in tissues, we used an analytical method based on the methodology used in the National Residue Control Plan for monitoring residues of coccidiostats in Poland. The method was validated according to the criteria described in Regulation (EU) 2021/808 [23]. The concentration of analytes in samples collected during the experiment was calculated using an appropriate matrix-matched calibration curve. The accuracy of the results was checked by preparing a spiked sample at a known concentration in each batch. The calibration range of toltrazuril sulfone was prepared in the following scheme: for the analysis of samples taken on day 8, the experimental curve was prepared in the 500–50,000 µg/kg range. Afterwards, the range of the calibration curve was set to 25.0–5000 µg/kg.

### 2.4. Data Analysis

The one-way ANOVA test was used to evaluate the differences in residue concentration and growth performance between healthy and experimentally infected birds. A value of *p* < 0.05 was considered to be statistically significant. Separate comparisons were made for liver and muscle samples.

## 3. Results

### 3.1. Growth Performance

The lowest weight gain was noted for chickens in experimental group B, which consisted of birds infected with *Eimeria* spp. and not treated with toltrazuril. Only results obtained from group B from day 8 of the experiment were consistently lower when compared to other groups. On the last day of the experiment, the mean weight gain of all groups improved compared to that of the control group except for group B. The weight gain of chickens during the experiment was analyzed by one-way ANOVA with Tukey’s HSD test. Post hoc comparisons using Tukey’s HSD test confirmed that the results for group B were significantly different from those of the other groups. Details of the results are shown in Table 2.

During sampling, the most severe changes in the small intestine and caecum were discovered in birds from group B: damage in the mucosa and in the lumen of the small intestine and severe hemorrhagic inflammation. Chickens in group B were also affected by the highest overall mortality. Five birds were found dead on day 5—four days after the *Eimeria* infection was performed, which was the most likely cause of the death. The influence of coinfection—*Eimeria* spp. and *C. perfringens*—caused a lower mortality rate: only one bird (which translates to 7% of the mortality rate) was found dead during the experiment. In chickens from this group, similar signs of coccidiosis were also observed. The mortality rate in groups where toltrazuril was administered was much lower: in groups D, E, and F, the mortality rate equaled 3%, 7%, and 0%, respectively.

### 3.2. Residues of Toltrazuril and Metabolites in Tissues

#### 3.2.1. Liver

- Toltrazuril Sulfone

**Figure 2:**
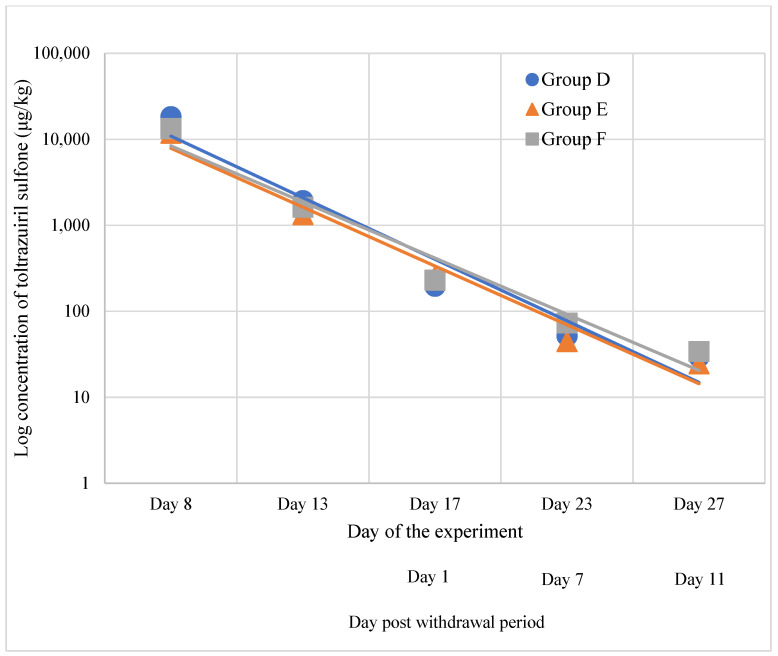
Depletion pattern of toltrazuril sulfone in liver samples collected in groups D, E, and F.

The highest concentrations of toltrazuril were found in group D samples, equal to 21,400 ± 5400 μg/kg. Samples collected from challenged groups were lower and equal to 11,770 ± 1090 μg/kg and 11,370 ± 2420 μg/kg for the *Eimeria* spp. group, and *Eimeria* spp. and *Clostridium perfringens* group, respectively. The results of the ANOVA test showed statistically significant differences in the concentration of toltrazuril sulfone in the liver (*p*-value < 0.05). Post hoc comparison using Tukey’s HSD test indicated group D as significantly different. Concentrations found in the challenged group were lower by 45% and 47% compared to group D. Along with the duration of the experiment, the residues of toltrazuril sulfone in liver samples decreased quickly. On the last day of the experiment, the residues of toltrazuril sulfone were as follows: group D: 47.3 ± 16.0 μg/kg; *Eimeria* spp. group E: 25.2 ± 11.6 μg/kg; and *Eimeria* spp. with *Clostridium perfringens* group F: 34.1 ± 11.7 μg/kg (Figure 2).

- Toltrazuril and Toltrazuril Sulfoxide

**Figure 3:**
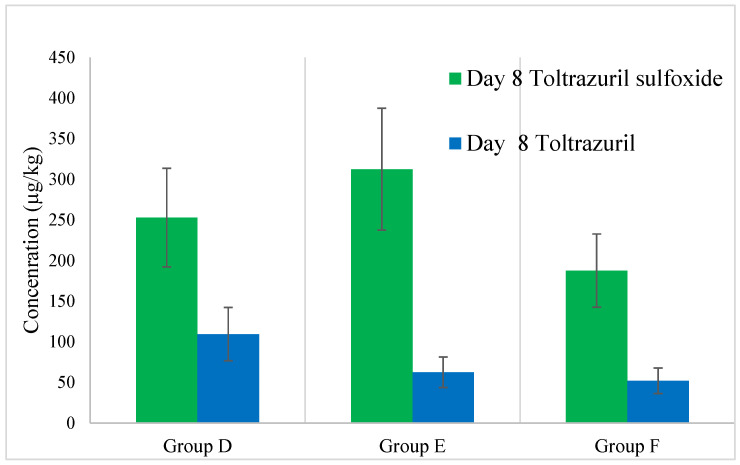
Concentration of toltrazuril and toltrazuril sulfoxide found in liver samples.

The concentration of toltrazuril and toltrazuril sulfoxide was significantly lower than the marker residue—toltrazuril sulfone. The highest amount of toltrazuril sulfoxide was found in group E on day 8 of the experiment, namely, 312 ± 129 μg/kg, followed by group D with 253 ± 62 μg/kg and group F with 188 ± 74 μg/kg. The toltrazuril concentration found in liver samples was even lower than that of toltrazuril sulfoxide. The highest residue concentration was observed in group D at a level equal to 110 ± 32 μg/kg. No statistical significance was found in the concentration data of toltrazuril (*p*-value = 0.1) or toltrazuril sulfoxide (*p*-value = 0.16). In samples collected after day 8 of the experiment, no residues above 25 μg/kg (limit of qualification) were detected. The graphical presentation of the concentration of toltrazuril and toltrazuril sulfoxide is shown in Figure 3.

#### 3.2.2. Muscle

- Toltrazuril Sulfone

**Figure 4:**
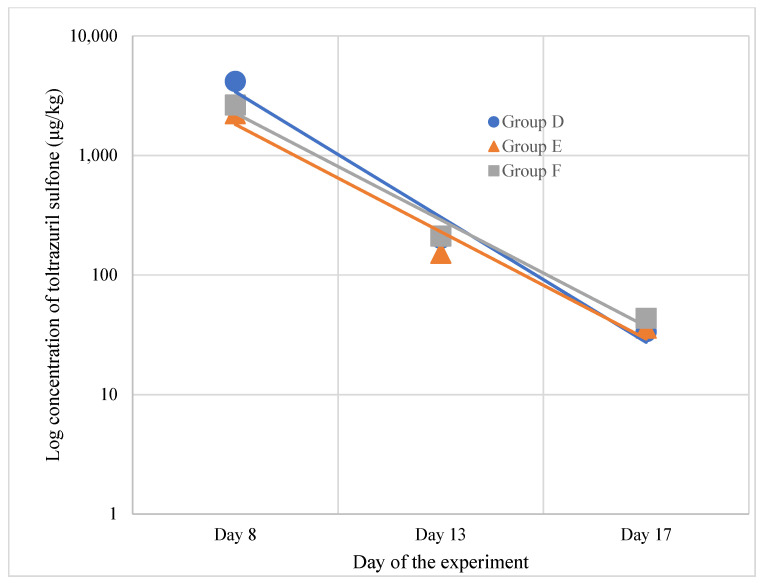
Depletion pattern of toltrazuril sulfone in muscle samples.

A similar pattern for toltrazuril sulfone residues was observed for muscle samples, with the main difference being a lower concentration compared to liver samples. In samples collected on day 8, the concentration in group D was equal to 4174 ± 1024 μg/kg, for *Eimeria* spp. group E, it was 2240 ± 515 μg/kg, and for the dual-infected group F, it was 2641 ± 874 μg/kg (Figure 4). The concentration in muscle was found to be statistically significant (*p*-value = 0.008). Post hoc comparison indicated results from group D to be significantly different.

- Toltrazuril and Toltrazuril Sulfoxide

**Figure 5:**
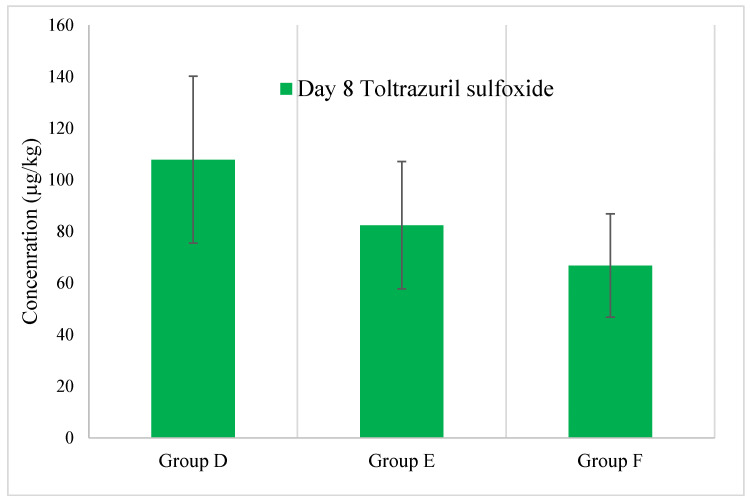
Depletion pattern of toltrazuril sulfoxide in muscle samples.

The pattern of lower concentration in muscle samples compared to the liver was also observed for residues of toltrazuril sulfoxide. The highest residue of toltrazuril sulfoxide was found in group D: 110 ± 32 μg/kg (Figure 5). No statistical significance was found in the concentration data of toltrazuril (*p*-value = 0.40) and toltrazuril sulfoxide (*p*-value = 0.17). No residues of toltrazuril were found in the collected muscle samples.

## 4. Discussion

### 4.1. Growth Performance

In our study, chickens challenged with *Eimeria* spp. and not treated with toltrazuril showed significant differences in average weight at the end of the experiment. Even due to the limited number of data points regarding growth performance in the present study, the results are in line with data published in the literature. Lower relative body weight gains of up to 20% as a result of coccidian infection (challenged with *Eimeria maxima* at a dosage of 5.0 × 10^3^ oocysts/bird) were reported by Lu et al. [24]. Mathis et al. also reported a lower mean of average weight for birds exposed to Eimeria parasites [25]. The analysis of results in groups with dual infection revealed no significant impact on weight gain. Initially, chickens presented lower weight gain, but to a lesser extent than chickens infected only with *Eimeria* spp. According to several sources, the presence of *Eimeria* spp. infection can be a predisposing factor for *Clostridium perfringens* proliferation [26,27,28]. The presence of *Eimeria* spp. parasites and their influence on the gastrointestinal tract can cause a large shift in microbiota composition and promote *Clostridium perfringens* replication. In a study by Lu et al., a dual infection scheme with *Eimeria* spp. and *Clostridium perfringens* was used to study body weight changes [24]. Similarly to our findings, the results on weight gain for the dual-infected group showed no significant differences compared to birds infected only with Eimeria oocysts [24].

### 4.2. Residues of Toltrazuril and Metabolites in Tissues

Depletion studies on toltrazuril were evaluated and published in the Toltrazuril Summary Report prepared by the European Medical Agency [16]. The report describes a study in which an undisclosed number of healthy chickens received toltrazuril at doses equal to 14.1 mg/kg of body weight per day on two consecutive days per week, three times at one-week intervals. The residues of toltrazuril and metabolites were analyzed in the following matrices: muscle, fat, skin, liver, and kidney. Residues of toltrazuril one day after the treatment were as follows: muscle 342 µg/kg and liver 870 µg/kg. Six days after dosing, no residues of toltrazuril above 25 µg/kg were found. In the case of toltrazuril sulfoxide, concentrations found on day one after the treatment were higher than those of toltrazuril—in the muscle, they were equal to 773 µg/kg, and in the liver, they were 3416 µg/kg. Similarly to toltrazuril, concentration on day 6 after the treatment declined quickly. No residues above 25 µg/kg were found in muscle, and in the liver, residues at a level of 54 µg/kg were found at this data point. The highest concentration in this study was found for residues of toltrazuril sulfone. On day one, the analyte was found in the muscle (4742 µg/kg) and the liver (21,275 µg/kg). Residues were found for a much longer period; even on day 20 after the experiment, 117 µg/kg was found in the liver, and in the muscle, concentration ranged from 30 to 85 µg/kg. The Summary Report also suggests setting maximum residue levels for toltrazuril sulfone as a marker residue of toltrazuril. The limits for chickens were recommended as follows: muscle—100 µg/kg; skin and fat—200 µg/kg; liver—600 µg/kg; and kidney—400 µg/kg, with a 16-day withdrawal period. Those recommendations were implemented in Commission Regulation 37/2010 [17]. Although the concentration found in our study cannot be directly compared to the EMA study due to different dosage schemes, a similarity in the rates of depletion of individual analytes can be observed between the two experiments. The studies on the metabolism of toltrazuril show that it is very well absorbed by the gastrointestinal tract and metabolized to short-lived toltrazuril sulfoxide and toltrazuril sulfone, which is considered a marker residue [29].

In a study published by Soliman, one-month-old chickens were medicated with toltrazuril at a dosage of 7 mg/kg of body weight (intra-crop-administrated) for 2 consecutive days [30]. Afterward, samples of muscle and liver were collected for 15 days. In this study, only residues of toltrazuril were analyzed. In liver samples, the highest concentration was found on day 1 after the medication, equal to 4700 ± 30 µg/kg. However, it declined rapidly and no residues were noted after day 8. In the case of breast muscle samples, the maximum concentration was 2600 ± 70 µg/kg. No residues were found after day 4 of the experiment. The results of this study revealed a surprisingly high concentration of toltrazuril in the collected samples. However, the depletion rate of toltrazuril is in line with other published studies.

In a study performed by Kandeel, the bioavailability of amoxicillin and ampicillin was evaluated in chickens infected with *Eimeria tenella* [31,32]. The results of those experiments showed an extensive decrease in the bioavailability of those drugs caused by major lesions and inflammation observed as the effect of coccidiosis. In conclusion, dose adjustment was recommended during the oral administration of both amoxicillin and ampicillin in chickens infected with cecal coccidiosis. Similar conclusions were formulated by Haritova et al. in their study regarding sulfachloropyrazine in chickens infected with *Eimeria tenella* [33]. They observed that the pharmacokinetics of sulfachlorpyrazine were significantly affected by the clinical and biochemical changes during coccidiosis in chickens. The rate of absorption of the drug was significantly decreased in infected birds. The authors also suggest that a longer withdrawal time can be expected in infected chickens. Interestingly, in this study, the concentration of sulfachlorpyrazine in the liver and intestinal wall was higher in infected poultry than in healthy poultry. The authors propose that the impaired integrity of the intestines contributes to higher drug penetration in the intestinal cells and higher absorption.

Because toltrazuril acts topically in the GIT, impaired absorption is not critical for pharmacological action. However, it was important to verify whether coccidiosis and/or *Clostridium* infection affects the level of residues. We found that it lowers the risk of residues of toltrazuril and its metabolites in edible tissues.

## 5. Conclusions

The impact of challenging factors on growth performance was observed only in chickens infected with *Eimeria* spp.—30% lower body weight compared to the control group. The influence of the parasite was also seen in lower concentrations of residues of toltrazuril sulphone in liver and muscle samples. The results obtained in this work confirm the influence of infectious factors on growth performance and residues. Furthermore, the results highlight the need for further research into how infectious factors influence additional performance parameters, such as feed intake and feed conversion efficiency, as well as the residues of coccidiostats in other tissues. Expanding the scope of such investigations will provide a more comprehensive understanding of how infections alter drug metabolism and their broader impact on animal production systems.

## Figures and Tables

**Table 1:** Description of animal groups used in experiment.

Experimental Factor	*Eimeria* spp.	Clostridium Perfringens	Toltrazuril
Day of the experiment/ Day of life	1/ 22	4/ 25	5 and 6/ 26 and 27
Group A—10 chickens	-	-	-
Group B—15 chickens	+	-	-
Group C—15 chickens	+	+	-
Group D—30 chickens	-	-	+
Group E—30 chickens	+	-	+
Group F—30 chickens	+	+	+

**Table 2:** Results of weight gain during experiment. Data are presented as mean ± SEM (*n* = 5). All weights in grams.

	Day of the Experiment						
	−21	1	8	13	17	23	27
Group A	38.7 ± 3.1 ^a^	132 ± 18.9 ^a^	184 ± 31.2 ^a^	206 ± 33.9 ^a^	261 ± 37.1 ^a^	281 ± 31.1 ^a^	301 ± 21.6 ^a^
Group B	42.4 ± 4.2 ^a^	103 ± 14.9 ^a^	110 ± 13.2 ^b^	128 ± 22.9 ^b^	142 ± 25.2 ^b^	161 ± 22.6 ^b^	182 ± 31.8 ^b^
Group C	41.2 ± 4.1 ^a^	125 ± 17.4 ^a^	162 ± 14.3 ^a^	192 ± 23.3 ^a^	223 ± 30.1 ^a^	279 ± 42.2 ^a^	299 ± 51.2 ^a^
Group D	41.9 ± 3.2 ^a^	129 ± 30.1 ^a^	182 ± 40.8 ^a^	212 ± 60.1 ^a^	251 ± 55.8 ^a^	291 ± 55.5 ^a^	302 ± 36.2 ^a^
Group E	39.4 ± 3.5 ^a^	132 ± 21.9 ^a^	153 ± 31.7 ^a^	182 ± 53.1 ^a^	249 ± 41.8 ^a^	271 ± 47.8 ^a^	301 ± 63.6 ^a^
Group F	42.5 ± 3.6 ^a^	136 ± 21.8 ^a^	162 ± 22.1 ^a^	191 ± 37.8 ^a^	249 ± 36.9 ^a^	281 ± 48.1 ^a^	302 ± 62.1 ^a^

The different lowercase superscript within a column indicates significant difference (*p* < 0.05).

## Data Availability

Data is contained within the article.
